# Using Smartphone Apps to Promote Psychiatric Rehabilitation in a Peer-Led Community Support Program: Pilot Study

**DOI:** 10.2196/10092

**Published:** 2018-08-15

**Authors:** Nora E Mueller, Trishan Panch, Cathaleene Macias, Bruce M Cohen, Dost Ongur, Justin T Baker

**Affiliations:** ^1^ Psychotic Disorders Division McLean Hospital Belmont, MA United States; ^2^ Wellframe Inc Boston, MA United States; ^3^ Department of Psychiatry Harvard Medical School Boston, MA United States

**Keywords:** psychosis, smartphone, app

## Abstract

**Background:**

Management of severe and persistent mental illness is a complex, resource-intensive challenge for individuals, their families, treaters, and the health care system at large. Community-based rehabilitation, in which peer specialists provide support for individuals managing their own condition, has demonstrated effectiveness but has only been implemented in specialty centers. It remains unclear how the peer-based community rehabilitation model could be expanded, given that it requires significant resources to both establish and maintain.

**Objective:**

Here, we describe the results from a study of one such program implemented within Waverley Place, a community support program at McLean Hospital, emphasizing psychiatric rehabilitation for individuals with severe and persistent mental illness, as well as describing the challenges encountered during the implementation of the program. Key questions were whether the patients could, and would, successfully use the app.

**Methods:**

The smartphone app offered multiple features relevant to psychiatric rehabilitation, including daily task lists, activity tracking, and text messaging with peer specialists. A 90-day program of activities, goals, and content specific to the community support program was created on the basis of a prior pilot, in collaboration between members of the app development team (WellFrame), and peers, clinical, and research staff associated with the program. Hospital research staff recruited patients into the study, monitored peer and patient engagement, and handled all raw data acquired from the study.

**Results:**

Of 100 people approached for the study, a total of 13 provided consent, of which 10 downloaded and used the app. Two patients were unable to complete the app installation. Five used the app regularly as part of their daily lives for at least 20 days of the 90-day program. We were unable to identify any specific factors (eg, clinical or demographic) that affected willingness to consent or engage with the app platform in the very limited sample, although the individuals with significant app use were generally satisfied with the experience.

**Conclusions:**

Smartphone apps may become a useful tool for psychiatric rehabilitation, addressing both psychiatric and co-occurring medical problems. Individualizing functions to each patient and facilitating connection with a certified peer specialist may be an important feature of useful apps. Unlike prior reports emphasizing that patients with schizophrenia will adopt smartphone platforms, we found that implementation of digital tools into existing community support programs for severe and persistent mental illness has many challenges yet to be fully overcome to realize the potential benefits such apps could have to promote systematization and cost reduction for psychiatric rehabilitation.

## Introduction

The imperative for health care providers to engage patients as proactive partners in their own health care has never been greater. A wide variety of community-based programs for people with mental illness are now recognized as evidence-based practices, but their provision of care is usually limited to the program facility because it is cost-prohibitive for program staff to meet with patients in their homes, neighborhood locations, or work settings [1-4]. This limitation in the loci of service provision is problematic because, while the psychiatric symptoms of many people can be symptomatically controlled by medication and psychotherapy, they are often not able to recover to premorbid levels of functioning without additional support in the community [2]. Therefore, there is a need for a cost-effective, low-effort measure to bridge this gap in service.

Mobile technologies, such as smartphone apps, show promise for remotely guiding and supporting patients with mental illness on an ongoing basis in a convenient and cost-effective manner. Smartphones, mobile phones that are able to access the internet and use apps, have become ubiquitous in the United States, and statistics indicate that this holds true among people with severe mental illness [5-8]. Additionally, previous studies have determined that mobile apps are acceptable to end users [9]. A systematic review by Firth et al found that in studies of smartphone apps in patients with schizophrenia, there was overall high retention and use by participants, with an overall retention rate of 92% [10]. The review also found relatively high use of the smartphone apps by participants, with participants using the apps more than 85% of the time [10]. In a previous study by Ben-Zeev et al, a smartphone app designed to provide illness management support to people with schizophrenia was used effectively by patients [11]. Participants were able to log on and access the resources on the app, participants accessed the app on average five times a day and 62% used it to reach out to their treaters [11]. In some studies, clinician engagement rates have been shown to be lower than patient rates [12]. One app designed for middle-aged people with serious mental illnesses, which led them through a ten-session psychosocial intervention on their smartphone, found good acceptability of smartphone app use in this population [13]. Additionally, the absence of stigma around use of smartphone apps may be one advantage of this approach. Previous research has determined that smartphones are viewed as a nonstigmatizing way to deliver care as they are commonplace in mainstream society, as is the use of mobile apps [14].

Despite progress, it is not clear whether older adults diagnosed with a major mental illness, many of whom also have a chronic physical health condition, are capable of using a health-promotion smartphone app on a regular basis in the community and if they are willing to do so. The present study examined the acceptability, viability, and usage patterns of a smartphone app developed by a software company (“WellFrame”), with which one of the authors is affiliated (TP). The app was tailored to the clinical programming of a community support program affiliated with McLean Hospital. A previous study of found that the smartphone app was acceptable to the program’s clients during a 30-day trial [15]. During that trial, all participants were successfully able to use the app, used the app an average of 94% of days in the study, and said they would continue to use the app if given the opportunity. In the present study, the trial period was extended to 90 days in order to determine whether individuals with severe and persistent mental illness would continue to sustain use of the app over a longer trial and whether acceptability and usage patterns would change, particularly given that usage of most apps drops off precipitously over time [15].

## Methods

### Study Sample

The site for this study was Waverley Place, an outpatient community support program for adults with mental illness affiliated with McLean Hospital. The majority of program members were aged 50 years or older, and most were diagnosed with a schizophrenia spectrum disorder or major affective disorder as a young adult. Daily activities offered by the program include mental health and addiction support groups co-led by staff and members, art and craft sessions, cooking classes, recreational and social activities, physical health promotion (exercise classes, trips to a local gym), counseling, and direct help with daily living tasks, as well as referrals to volunteer or paid work outside the program. Additionally, every member chooses a “contact person,” a certified peer specialist, with whom they check in on a semiregular basis. The program employs both Master’s level mental health staff and certified peer specialists. The program administrator is a psychiatrist at McLean Hospital.

#### Recruitment and Screening

This protocol was reviewed and approved by the Partners Healthcare Institutional Review Board and all participants gave informed consent. A convenience sample was recruited during usual group activities at the program, for example, support groups, program planning meetings, exercise sessions, social events, and impromptu gatherings for casual conversation and recreation. Typically, a study representative provided a brief description of the smartphone app (“*WellWave* ”) and the app evaluation study, inviting those interested in learning more to meet with the study representative individually at a later time, where they demonstrated the app and explained study aims and procedures. Additionally, the study staff attended all community meetings to make announcements about the project and posted flyers with study staff contact information in order to stimulate recruitment. A total of 100 individuals were approached by research staff, over an 8-month period of enrollment. To ensure inclusiveness, the only eligibility criteria for study enrollment were (1) access to a mobile phone compatible with the app or willingness to use a study-provided one and (2) current membership in Waverley Place. Interested individuals who did not own an app-compatible phone were told they could receive a loaner phone as soon as these were available, and that the project would provide instruction in how to use the phone. Only 1 participant accepted a loaner phone.

### Smartphone App Intervention

This pilot study focused on the two main components of the *WellWave* app: (1) to improve patient functioning and (2) to allow program staff to confidentially monitor the well-being of patients when they are not attending the program. The purpose of the app is to improve engagement of individuals in the rehabilitation program through increasing communication with their care provider and assigned peer specialist. A detailed description of the development and initial pilot of the app are reported by Macias et al [15]. Members of the Wellframe staff were actively involved in the planning and support phases of this study and provided the app free of charge for the duration of the pilot, as well as providing the phones for participants without smartphones to use. Members of the WellFrame staff did not, however, engage in any direct communication with the study participants, either in person or online, at any time during the study procedures.

#### Daily Tasks Lists

The *WellWave* app generates a list of suggested activities for each day in the 90-day study period using a predefined sequence that was fixed for all participants, such that a new “Daily Tasks” list would appear on the participant’s phone screen every morning. These optional activities included internet links to recommended readings and videos; questionnaires including brief free-text reports on well-being (eg, “I am feeling...”) or mood and symptom self-ratings; and exercise prompts, which consisted of a goal for daily walks (ie, steps or time-based) that gradually increased from 5 minutes to 20 minutes over the course of the 90-day program. Medication reminders also appeared on the Daily Tasks list for participants who choose to set up this option. An exercise reminder appeared on the screen every day. Testimonial or motivational videos created by the community support program’s current peer staff appeared on the Daily Task list early in the 90-day program to encourage engagement.

The app automatically recorded each participant’s selection of a task, how long the selected task remained open, and any response the participant made to the task prompt. These recordings were subsequently used to graph fluctuation in task engagement over time. For instance, the app electronically tracked which internet resources (eg, psychoeducational videos and readings) were activated, as well as the duration of engagement.

In addition, participants were asked to report (through the app) their emotional and physical well-being; relative ease of use, which could be visualized in a Web-based dashboard visible to the research and program staff; completion rates; and sensitivity to detect change over time in relation to life events reported by each user. These self-report measures included standardized self-assessment instruments, including brief versions of the PROMIS scales [16], single-item global questions commonly used in medical settings (eg, “How would you describe your physical health?” where 1=poor to 5=excellent), and brief mood and symptom checklists. A total of 2 to 6 tasks appeared on the app’s Daily Tasks screen on any given day.

#### Text Messages

The *WellWave* app offered two-way text messaging between app users and community program peer staff (certified peer counselors diagnosed with a mental illness). This messaging was confidential and HIPAA-compliant because all text conversations took place exclusively within the app. The app preserves this text, ordered by sender id, date, and time of day. In this pilot study, we were primarily interested in measuring how often such electronic contact took place, and how often the messaging was reciprocal (ie, a two-way conversation between peer staff and app user).

### Measures

#### Intake and Exit Interviews

Questionnaires were administered at the time of enrollment and in a poststudy exit interview. Intake questions were designed to measure (1) prior experience with smartphones; and (2) current mental and physical health problems that might interfere with app use. The latter measure of self-perceptions used the same wording and scaling as measures in the *WellWave* app. Questionnaires were offered as a choice between paper or computer formats, which used REDcap. The exit interview questionnaire also contained several rating scale measures of satisfaction with the app, and inquired about personal reasons for using or not using the app. The app feedback questionnaire was posed as a series of prompts, such as “I still find it hard to use WellWave without asking for help” and “I'd recommend WellWave to a friend” rated on a 1 to 5 Likert scale. A member of the research team also conducted interviews with each of the participants during the exit interview. This interview consisted of 12 questions about specific features the participants liked and didn’t like about the app, and whether or not they would continue the app if given the option. 

#### Engagement With the Smartphone App

Ease of use and acceptability were measured quantitatively, including items for whether app training was successfully completed (yes or no), how frequently the app was used, how intensively each of the app components was used, and duration of app use during a common 90-day study period.

*Frequency of app use* was operationally defined as a count of study days the app was used (turned on) during each participant’s 90-day study period.

*Intensity of app use* was defined as the ratio of total tasks opened to total tasks viewed in the Daily Tasks list across all days the app was used. *Intensity* was calculated for each participant as one summary percentage, and as separate percentages for specific types of tasks. Because only one participant chose to use the medication reminder option, summary measures of use *frequency* and *intensity* were based only on prompts in the Daily Tasks lists that reminded participants to exercise, offered links to videos and readings, or asked for reports of well-being.

*Duration of app use* was defined as a count of calendar days between first and last day the app was used by each study participant.

## Results

### Participants

**Figure 1:**
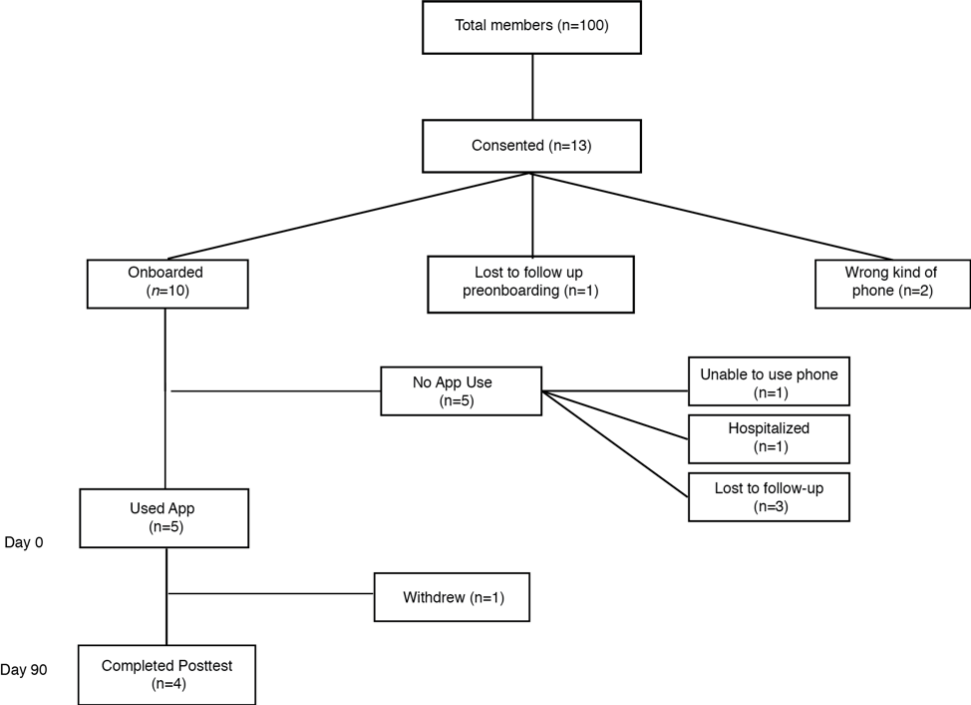
Outline of participant flow through the study.

Figure 1 outlines the participant flow through the study. Of the 100 people eligible and approached to participate in the study, a total of 13 were recruited. Members who met study eligibility criteria entered the project gradually over an 8-month open enrollment period (August 31, 2015 to March 4, 2016). However, only ten participants actually downloaded the app and were onboarded to the study. Of the three that never downloaded the app, 2 were excluded due to incompatibility of their personal smartphone; and 1 was lost to follow up after consent. Of the remaining 10 participants two individuals were unable to use the app after intake training. Three successfully completed app training, but then used the app only briefly. Five participants used the app on 20 or more study days (range 20-92 days). All 13 participants completed baseline assessments and were therefore included in analyses of baseline data.

All the participants were non-Hispanic Caucasian. A majority were over the age of 40 years (n=8), and about half were over the age of 50 years (n=6). Most of the participants were women (n=10). The women were more diverse in age (range 29-63 years), and the men were aged 52-60 years. The sample was split between those diagnosed with a schizophrenia spectrum disorder (n=5), and those with a major affective disorder (n=8), either bipolar disorder or severe depression. Most of the sample (n=7) also reported being under treatment for a chronic physical condition (eg, heart disease, asthma, arthritis, cancer). Psychiatric and physical health diagnoses were evenly distributed across age groups and sexes. As is true of the program membership in general, the study sample was well-educated: all except 1 study enrollee had some postsecondary education, and 9 of the 10 had completed 2 or more years of college. Two of the 10 were already in a previously published pilot [14].

The following case descriptions of study participants include identified reasons for not using the app, and explicit or inferred attitudes expressed by participants toward the app. These qualitative explanations for app use or nonuse were derived from researcher-recorded observations during and after app training, participant verbal and written feedback in exit interviews, text messages sent to staff regarding app use, and participants’ free-text responses to app-generated questions about their health and daily activities. Additionally, participants total messages received and sent are displayed in Table 1.

#### Unable to Use App

Two participants (both women) who were unable to use the app at all after training. One (aged 61 years, bipolar disorder) had difficulty staying awake during app training, was hospitalized for psychiatric reasons a few days later, and remained hospitalized for the duration of the study. The other (aged 63 years, schizophrenia) accepted a loaner phone in order to be eligible for the project, but she had minimal experience using a smartphone, and after 3 instructional sessions, gave up trying to learn to use the app. Both participants had earned college degrees (Master’s degree and registered nurse) as young adults, were long-term members of Waverley Place, and checked psychotic symptoms and cognitive problems on intake questionnaires.

#### App Training Only

One man (aged 52 years, major depression) and two women (aged 24 years, bipolar disorder and aged 59 years, schizophrenia) completed intake training but only used the app for at most five days. All 3 were relatively new to the community support program. In their exit interviews, the man (age 52 years) said, “I forgot I had the app.” The older woman (age 59 years) said she had accidentally deleted the app. The 24-year old woman was lost to follow-up soon after enrollment.

#### App Engaged

Of the 13 participants recruited, 5 study participants used the app for 20 or more of their 90 study days. Observations made by study staff and program staff about individual participants utilization of the app are described in Table 2.

### Associations Between Participant Characteristics and Level of App Engagement

The sample was homogeneous with regard to race (all non-Hispanic Caucasian), education (12-16 school years), prior experience using a mobile phone (all except one had used a smartphone for 2-4 years), and current level of exercise (all had some scheduled physical activity). Due to the small sample size we could not test whether any of these four background characteristics had any impact on app use. However, from a general examination of the data there does not seem to be any associations.

### Participant Feedback

Five participants completed an exit interview at the end of the 90-day study period. In contrast to an earlier 30-day pilot study, where all participants reported satisfaction with the app [14], in the present study only 2 participants said they would continue to use the app if given the chance to do so. Although, one participant who reported that they would not want to continue using the app, cited frustration with the technical malfunctions of the exercise portion of the app, where the exercise tracker would stop working mid-walk, as their main source of dissatisfaction and stated that “...without the exercise part, the videos were good, and the med reminders were good.” Additionally, 4 of the 5 participants indicated that they enjoyed receiving messages from the peer specialists and that these messages made them feel more connected to the staff. Participant’s suggestions for improving the app included adding a social feature to interact with other members.

**Table 1:** Frequency of messaging of participants and staff members.

Subject ID	Number of user messages	Number of staff messages
WW013	0	10
WW012	1	7
WW010	0	11
WW009	5	23
WW007	0	14
WW006	2	23
WW005	14	34
WW004	0	0
WW002	4	16
WW001	53	51

**Table 2:** Case descriptions for the 5 participants who actively used the app beyond onboarding.

Characteristics (age, gender)	Diagnosis	Key app use features	App use difficulties
Late 40s, female	Schizophrenia	While active, they engaged in almost all the app digital components (eg, surveys, videos, readings) except medication management, and took 20 app-recorded walks during her 28 days of app use.	Hand tremors made it difficult to key responses into their phone, but they did not mention this as an obstacle to app use. The reason they gave for withdrawing from the project was that the app surveys designed to track her moods and psychiatric symptoms “made me feel bad” and “more symptomatic.”
Late 20s, female	Schizoaffective Disorder	This participant used the app about half the days she was enrolled in the project, and her days of use were sporadic across the 90-day study period.	They refused to complete both intake and exit questionnaires. The participant took only 3 recorded walks and expressed concern at intake that her exercise performance would be compared to that of other participants.
Early 40s, female	Bipolar Disorder	This participant remained steadily engaged in the app for the duration of the study in spite of several chronic health conditions (cardiovascular disease, diabetes, asthma), recurrent migraine headaches, and an acute episode of severe bronchitis. Their primary psychiatric symptoms were anxiety and depression, and she rated herself at intake as very lonely, suspicious, edgy, and easily upset. The participant reported that insomnia often left them tired and unable to concentrate, but they used the app almost daily, and responded to about half of the tasks they saw listed on the Daily Tasks screen. They also had the second-highest rate of conversational text-messaging with peer staff, and the second-highest count of app-recorded exercise (31 walks).	Not applicable.
Early 60s, male	Schizophrenia	This participant opened the app nearly every day to see what was listed in the Daily Tasks screen. They were also intensively engaged in all the app tasks except exercise (8 recorded walks). The participant responded to 56% of the tasks he viewed on the Daily Tasks list, completing about 70% of the app-generated surveys, and connecting to 70% of the internet readings and videos. The participant sent a text to peer staff saying “This is a good app!”	They exhibited hand tremors during app training and reported that sleep apnea reduced his ability to concentrate.
Early 60s, female	Bipolar Disorder	By the end of the project, this participant had viewed 90% of the internet readings and videos that appeared on the Daily Tasks lists, completed 84% of the self-report surveys, and they were the only participant to use the medication reminder option. In spite of needing a walker to get around, they recorded 58 walks on an elliptical machine in response to exercise prompts. This participant also responded to almost all the self-report surveys received on their phone and sent periodic free-text descriptions of events in their social life.	This participant was slow in keying in responses because of hand tremors.

## Discussion

### Principal Results

The aim of this study was to determine if extending the trial of a previously piloted smartphone app from 30 days to 90 days would affect the acceptability and use patterns. In the present study the uptake of the app was low: of the 100 members of the program approached to participate in the study, only 13 signed consent and only 10 downloaded the app onto their phone, which was not a problem encountered in the previous study. However, those who did use the app found it acceptable.

The current study is notable because of the recruitment difficulties encountered by the study staff. Despite the minimal inclusion criteria and efforts of the research staff to use multiple avenues to stimulate recruitment (one-on-one recruitment, flyers, group announcements) only 13 members of the 100 approached ultimately consented to be in the study. Technology is often looked to as an exciting component for the future for physical and mental health care, but this study may be a demonstration that although conceptually mobile apps may provide a way to provide more or better services to people, and fulfill a need in health care, sometimes people do not want to use them. The present study was able to provide a better look at the desire for people to engage with these kinds of apps in a real-world situation than previous studies, because unlike in previous studies, participants were provided no monetary compensation for their participation [17]. Therefore, the participants had little other incentive to participate other than interest in the app itself.

There are certain characteristics of Waverley Place that may have contributed to the low adoption of the app in this population. Specifically, members of this day program already had a staff member who they were assigned to check in with them and provide support, reducing the need for an adjunctive digital support tool. Additionally, the day program is open 8 hours a day, 5 days a week and members are free to come in and receive support and guidance from a staff member at any time. Consequently, there may have been no need for this app to fill in this particular program. However, a similar app may do very well in a population where time and resources of the program and staff are more limited, and this app, or similar apps, may represent a way to maintain contact with members in ways they otherwise would not be able to. Therefore, as more smartphone apps are developed the target population should be carefully considered to determine whether a smartphone app would be wanted or useful.

People who are older may be less technologically literate, which was demonstrated in our sample by the difficulty study staff had teaching participants using study provided smartphones (several one-on-one sessions, before the participant ultimately withdrew). Participants in this study were older adults with over half being over the age 40, and this age range was representative of the program members, particularly given that those who attend the program on a regular basis are older, indicating that this may have limited the adoption and use of the app in our study. Additionally, many of the members of the program used government subsidized flip phones and did not want to use an additional device for the duration of the study, which is similar to results found in other studies in community support clinics [17]. Therefore, smartphone apps such as *WellWave* may be more successfully accepted in a population where people are already using a smartphone, such as in a population of younger adults, in an early intervention or first episode clinic. For example, in a study published by Kumar et al) a majority of their potential participant pool did not have a smartphone to begin with, but they were able to effectively able to distribute smartphones for use during their intervention, potentially due to using an Early Psychosis population [17]. Interestingly, Ben-Zeev et al was able to effectively train and deploy study supplied phones in their intervention, in a study with a mean age of 45.9 years, similar to that of the present study [11]. Therefore, there may be more factors impacting a person’s interest in using an app other than age, and technological literacy, which is commonly associated with age, that need to be explored in order to develop a strategy for the most effective development and implementation of these resources.

### Implications for the Design and Evaluation of Mobile Phone Apps

Smartphone apps that require interactive responses to multiple features appear to be appropriate for most adults with a major mental illness who have smartphone experience, 80% (8/10) of study enrollees who downloaded the app were able to learn to use the app, and 50% (5/10) found the app acceptable for regular use in their everyday lives. However, the personal reports suggest that use and comfort with specific features differs greatly among individual patients.

Participants who used the app regularly had moderate to high response rates for every app component, except the option to receive medication reminders. Most study enrollees had been coping with mental illness for many years, so it is likely they already had medication management systems in place and, unlike new patients, may not need these reminders. Alternatively, medication use is often a complex negotiation between patient and clinician, and an app may not be the best way to assist compliance for many users.

Smartphone apps, like *WellWave*, appear especially useful for community support programs dedicated to promoting autonomy and community living because patient well-being can be assessed frequently and efficiently, at low cost and with minimal staff time.

More research is needed to determine which app features are most important to adults with mental illness and their mental health service providers, as well as what level of app complexity is a good fit with particular groups of people who share the same psychiatric symptoms or educational attainment. For instance, the participant who withdrew after a month in the project said that she found the negatively-worded symptom-assessment questions particularly distressing. Ideally, smartphone apps should be pilot-tested continuously in small sample studies while they are still being developed so that app features can be added and tested in a stage-wise fashion, with one or two new components added at a time, beginning with a streamlined menu of app activities and progressing toward a more demanding design. This incremental approach would reveal at what level the app becomes unacceptable or unusable by people with differing levels of functioning and cognitive clarity and allow the eventual production of separate versions of the same app tailored to the needs of specific types of people. Beyond types and groups, symptoms, styles, and needs are very different from one person to the next, and app design and choice may need to be individualized.

Additional research should assess what factors impact the adoption of a smartphone app in a population. Apps and other technology are looked to as potential ways to disseminate quality mental health care more efficiently, but as the present study illustrates more work needs to be done to understand what interventions will work and will be accepted in a given population.

### Limitations

This study was limited by a small sample size, which may limit the generalizability of our findings to other clinical samples and treatment settings. Nonetheless, our experiences provide some unexpected insight into the demand for smartphone apps in a population like Waverley Place when no other financial incentive is given. The small sample size did not allow us to draw any conclusions about the participant characteristics that most influenced app use. Although some anecdotal data was collected by study staff about the reasons members of the program did not want to participate, further research is warranted to explore more systematically the reasons and characteristics of people who do and do not want to participate in these app studies. This would have important implications for development and dissemination of future apps to support individuals with severe and persistent mental illness.

### Conclusions

A smartphone app designed to promote and track emotional well-being can become a useful tool for many community-based patients served by mental health programs. All adults diagnosed with a major mental illness should be offered the opportunity to learn to use a health-promotion smartphone app even if they are psychiatrically symptomatic, past the age of 50 years old, or coping with a cognitive impairment or a disabling physical health condition. However, this study illustrates the importance of researchers being cognizant of factors that may affect the implementation of such apps in existing community support programs, such as how features in the app complement or overlap with existing services, and the extent to which the application is offered as a research opportunity versus an integral part of the program’s support services.
